# Are Information Technologies Capable of Stimulating the Use of Dental Floss by Adolescents? A Cluster Randomised Clinical Trial

**DOI:** 10.3290/j.ohpd.a44684

**Published:** 2020-07-04

**Authors:** Gisele Marchetti, Luciana Reichert da Silva Assunção, Geisla Mary Silva Soares, Fabian Calixto Fraiz

**Affiliations:** a Paediatric Dentist, Department of Stomatology, Universidade Federal do Paraná, PR, Brazil. Collected the data, interpreted data, wrote the manuscript.; b ^Associate Professor, Paediatric Dentistry, Department of Stomatology, Universidade Federal do Paraná, Curitiba, PR, Brazil. Responsible for study design, performed statistical analysis and interpretation of data, and critically reviewed the manuscript.^; c Adjunct Professor of Periodontology, Department of Stomatology, Universidade Federal do Paraná, Curitiba, PR, Brazil. Responsible for calibrating the clinical parameters, critically reviewed the manuscript.; d Titular Professor, Paediatric Dentistry, Department of Stomatology, Universidade Federal do Paraná, Curitiba, PR, Brazil. Research advisor, study design, statistical analysis, interpretation of data, critically reviewed the manuscript.

**Keywords:** adolescent, Information technology, oral health

## Abstract

**Purpose:**

To analyse the effect of information technologies on improving the frequency of the use of dental floss among adolescents.

**Materials and Methods:**

A randomised, controlled clinical trial was conducted with 291 adolescents (mean age: 16.1 years) in three phases. Phase I involved the application of a questionnaire and clinical examinations using the simplified Oral Hygiene Index and gingival bleeding index. In phase II, the adolescents were randomly allocated to four groups: oral counseling (OR) and the use of an application (App) for smartphones; OR without the app; video (VD) and app; and VD without app. Messages were set through the app for 30 days. Phase III involved the second administration of the questionnaire and clinical examination. The frequency of dental floss use was evaluated in phases I and III. The groups were categorised into the use of technology (VD and/or App) and non-use of technology (OR alone).

**Results:**

Statistically significant reductions in the clinical indices were found with all educational methods (p < 0.005) and improvements were found in the use of dental floss (p < 0.001). Moreover, information technologies were associated with an improvement in the frequency of dental floss use (p < 0.033).

**Conclusion:**

All methods were effective at improving clinical indicators. The use of information technologies can be considered an effective tool for improving dental floss use among adolescents.

Adequate oral hygiene habits are of particular importance in adolescence, as behaviours acquired in this period tend to perpetuate in adulthood.^6,19^ However, the specificities of adolescents make this group resistant to the adoption of adequate oral health habits,^20^ especially the use of dental floss.^28^ Changing such behaviour is a challenge for clinicians. Overcoming barriers requires knowledge regarding the characteristics of this phase of life and adequate communication strategies.

With the development of the internet, access to information has undergone revolutionary changes and information technologies have been redefined to incorporate this new web-based means of communication. In 2017, more than 90% of Brazilian adolescents used the internet and the portion of youths that access the web through mobile devices is larger than the portion that uses computers.^9^ This demonstrates the importance of mobile devices as a tool for facilitating access to information^7,17^ and points to the immense potential for the appropriation of channels of communication as education methods, especially for adolescents. Indeed, texts and applications for smartphones used as health education strategies for this age group have improved behaviours and increased the incorporation of preventive measures regarding human papillomavirus,^25^ the use of oral contraceptives,^7,31^ habits involving alcohol and tobacco,^15,16,27^ reductions in body fat,^11,12,22^ and improvements in oral hygiene and gingival bleeding indices.^24,32^

The use of these communication tools may improve oral hygiene habits among adolescents. Among such habits, the one with the least adherence is the frequent use of dental floss. Although important to the maintenance of gingival health, dental floss use is low among adolescents.^8,21,28,30^ Thus, the aim of the present study was to determine whether the use of information technologies is capable of improving the frequency of dental floss use among adolescents.

## Materials and Methods

This study was conducted in accordance with the precepts stipulated in the Declaration of Helsinki and received approval from the Human Research Ethics Committee of the Federal University of Paraná (process No. 51712315.4.0000.0102).

### Study Design and Sample

A randomised controlled clinical trial (clinicaltrials.gov, No. NCT03216746) was conducted involving male and female adolescents between 14 and 19 years of age at a public high school in the city of Curitiba, Paraná, Brazil. The exclusion criteria were physical or mental condition that impeded the interventions, absence of signed statements of informed consent (adolescent and guardian), use of fixed orthodontic appliance at the time of the examination, enrollment in technical courses related to the field of oral health, and refusal to participate in any phase of the study.

We used the sample from another study evaluating the influence of different educational methods on improvements in knowledge among adolescents regarding periodontal health,^24^ totaling 317 individuals.

### Pilot Study

A pilot study was performed to test and adapt the methods and instruments. This step was also used to determine the dynamics of the application of the instruments and mean time spent with each participant. For such, we selected 15 adolescents between 14 and 19 years of age with the same socioeconomic characteristics as the study population. These individuals did not participate in the main study. The methods and instruments were considered adequate and no changes to the initial proposal were deemed necessary.

### Questionnaire

A self-administered questionnaire (tested in the pilot study) with multiple choice questions was used to determine the use of dental floss before and after the educational actions. Dental floss use was investigated with the following question: “Do you use dental floss?” The response options were “yes, every day”, “yes, but not every day” and “never or rarely”. While answering the questionnaire, the students were supervised by one of the researchers to ensure no interpersonal communication. The guardians of the adolescents also answered a questionnaire addressing socioeconomic and demographic characteristics. The criteria of the Brazilian Association of Research Firms^3^ were used, which classifies families based on the possession of household appliances and items from a previously established list. The individuals were then categorised into five economic classes ranging from A1 to D-E.

### Interventions

**Fig 1:**
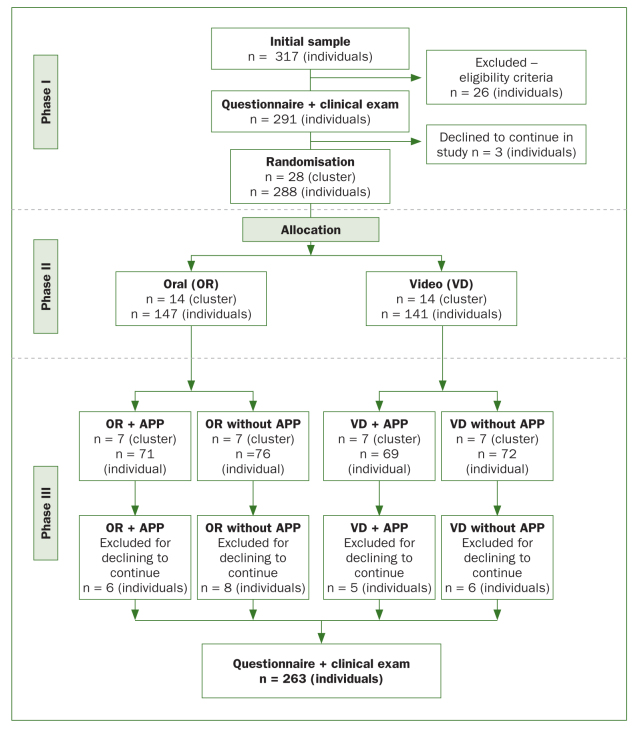
Flowchart of study.

The study was developed in three phases (Fig 1). In phase I (baseline), the participants (n = 291) answered a questionnaire addressing behaviour related to the use of dental floss and were examined clinically to determine the Simplified Oral Hygiene Index (OHI-S)^14^ and gingival bleeding index (GBI).^1^ In phase II (n = 288), the adolescents were randomly allocated to two educational interventions (oral counseling [OR] and video counseling [VD]) using a simple lottery system with sealed, opaque envelopes. Cluster allocation was performed, with the groups considered as the sampling units. The two groups were then divided into subgroups, two of which involved the use of a smartphone application (app): OR+app, OR without app; VD+app, and VD without app). After a period of 30 days, the participants (n = 263) answered the questionnaire again and were reevaluated clinically.

### Counseling

Oral counseling with a standardised content was performed by one of the researchers (GM) who had previously undergone training. Counseling addressed aspects of general health, oral health and, particularly, periodontal disease. This intervention lasted approximately 15 min and was conducted in the classroom with a group of approximately 20 adolescents, providing a setting for the discussion of the topics addressed.

The aim of the video was to offer a means of teaching likely to attract the attention of the target audience. The video involved the participation of three actors (two adolescents and one playing the role of dentist). The video had a length of 14 min and was shown in the classroom to a group of approximately 20 adolescents, but with no opportunity to discuss the topic afterwards.

An application called “Oral Health” was designed specifically for the present study. The application was developed for the Android 4.4 system (Kitkat) API level 19 in JAVA (JDK 1.8.0) in the Android Studio 2.1.2 development medium and had 27.90 megabytes. The application was developed free of charge in Google Play for 12,119 cellular phone models. This tool was designed to transmit oral health knowledge in a clear, accessible manner to the target audience over a 30-day period. To this end, 60 messages were developed based on the content of the previously described educational activities. Messages were sent twice per day to each participant: the first with written information and the second in video form. Each video lasted an average of 1 min and was developed specifically for the present study to reinforce the content of the preceding text message.

The participants received messages through the Android notification bar. The sound and vibration of the cellular phone were activated upon receiving the messages. To enable this, permission from the smartphone was needed to access the internet, vibrate the phone, keep the screen on while playing the video, and initiate the application upon switching on the cellular phone. The app executed its functions even with the smartphone offline, only requiring access to the internet while downloading the app. The source code of the application can be obtained from the GitHub address (https://github.com/willianmuniz/saudebucal) and can be accessed and used by any user.

### Clinical Examination

The clinical oral examination was performed by a researcher who had undergone a training and calibration exercise (κ ≥ 0.83) for the determination of dental biofilm using the OHI-S^14^ and GBI.^1^ The examiner was blinded to the allocation of the participants to the different groups. The examinations were performed in phases I and II in a classroom using gauze, artificial light, and a millimeter probe. Each participant was examined individually while sitting in a chair.

### Statistical Analysis

To analyse the use of dental floss, this variable was dichotomised as “every day” and “not every day/never”. The comparison between phases I and II was performed using the nonparametric McNemar test. For the comparison of the use of dental floss in relation to the different educational interventions, the groups were categorised into those with technology (VD and/or app) and without technology (OR alone). “Use of dental floss” was categorised as “improved or continued good” and “worsened or continued poor”. This analysis was performed using Pearson’s chi-squared test, with the estimate of prevalence ratios (PR) and respective 95% confidence intervals (CI). For the descriptive analysis of oral hygiene, the OHI-s was dichotomised as indicative of a high plaque index (OHI-s >1) or low plaque index (OHI-s ≤1). The Wilcoxon test was used to compare phases I and III with regard to the groups “with technology” and “without technology” and the clinical indices (OHI-s and GBI, analysed as quantitative variables). All analyses were performed with the aid of the SPSS v 25 (IBM; Armonk, NY, USA), with the level of significance set at 5% (p < 0.05).

## Results

Among the 291 adolescents recruited in phase I, 159 (54.6%) were girls, the mean age was 16.1 years (SD = 1.21; range: 14 to 19 years), the mean monthly family income was US$ 1023.22 (SD = 585.98), and the majority belonged to economic class “B” (77.8%). Regarding the frequency of dental floss use (Table 1), a statistically significant improvement was found when comparing the two evaluation times (p < 0.001): 140 (60.9%) of the 230 participants with inadequate use (not every day/never) in phase I began to incorporate the habit on a daily basis.

**Table 1:** Frequency of dental floss use before and after educational interventions (n = 263)

	Use of dental floss in phase III			
Use of dental floss in phase I	Every day	Every day n (%)	Not every day/never n (%)	Total n (%)
		28 (84.8)	5 (15.2)	33 (100)
	Not every day/never	140 (60.9)	90 (39.1)	230 (100)
	Total n (%)	168 (63.9)	95 (36.1)	263 (100)

p < 0.001 (McNemar test).

Table 2 displays the association between the use of dental floss and groups with or without technology. The use of technology was associated with an improvement in dental floss use (p = 0.033); individuals who received educational interventions involving the use of technology were 44% more likely to improve or maintain an adequate tooth flossing frequency compared to those without access to technology.

**Table 2:** Comparison between groups with and without technology according to improvement, worsening, or maintenance of oral health behaviour (n = 263)

	Group	Worsened or remained poor n (%)	Improved or remained good n (%)	Total n (100%)	p*	PR	95% CI
Dental floss	Without technology	33 (46.5)	38 (53.5)	71 (27)	0.033	1	1.042–1.988
	With technology	62 (32.5)	130 (67.7)	192 (73)		1.439	

* Chi-squared test. PR = prevalence ratio; CI = confidence interval. Statistical significance was set at p < 0.05.

Prior to the interventions (phase I), the prevalence of a high plaque index was 70.7%. In phase III, all participants (n = 263) had a low plaque index. Moreover, a statistically significant difference was found regarding the GBI, with a mean of 10.42 (SD = 4.38) in phase I and 2.12 (SD = 1.84) in phase III (p <0.01). Table 3 displays the mean OHI-s and GBI in phases I and III according to educational intervention (with and without technology). Statistically significant reductions were found in both groups (p < 0.001).

**Table 3:** Mean OHI-s and GBI in phases I and II according to intervention group (n = 263)

	Group	Phase I Mean (SD)	Phase III Mean (SD)	p*
OHI-s	With technology	1.24 (0.37)	0.25 (0.18)	< 0.001
	Without technology	1.35 (0.26)	0.26 (0.19)	< 0.001
GBI	With technology	10.64 (5.09)	1.99 (1.56)	< 0.001
	Without technology	9.86 (4.06)	2.48 (1.64)	< 0.001

* Wilcoxon test; statistical significance was set at p < 0.05.

## Discussion

The present study confirms that educational actions have a positive impact on clinical indices in adolescents and that the use of information technologies has the potential to increase the frequency of dental floss use in this population. These findings are important, as the daily control of dental biofilm is the most effective method for maintaining oral health.^26^ Although some studies have indicated that there is insufficient evidence to support the use of dental floss,^29,33^ daily use is recommended by several associations, such as the American Dental Association^2^ and the Australian Dental Association,^4^ Interproximal cleaning is considered to be an important aspect of oral self-care.^5^

The literature has shown that convincing individuals to use dental floss is challenging, and adolescents generally do not engage in this practice.^8,23,30^ A lack of motivation has been described as one of the reasons for low adherence to the use of dental floss among children and adolescents.^26^ Moreover, a prospective study involving 258 adolescents and adults found that motivation for the use of dental floss diminishes as soon as supervision ceases.^30^ The reason for this is the fact that attempts to modify behaviour have traditionally focused on the initial intervention, with little attention given to strategies for maintaining the behaviour. Thus, interventions cease when the target behaviour is obtained and the initial gains tend to disappear over time.^18^

Therefore, it is necessary to identify and test methods that can maintain behaviours over time and are acceptable to the young population. The results of the present investigation confirm the findings of previous studies reporting that more attractive educational methods are able to captivate youths and are more easily assimilated by this target population.^13^ Moreover, these tools are available to users at any time and can enhance the retention of knowledge, as demonstrated in a recent randomised controlled clinical trial that tested educational methods on Brazilian adolescents.^24^

The high indices of bacterial plaque and gingival bleeding prior to the interventions confirm that adolescence is a period of risk for the development of oral diseases, especially periodontal disease.^10^ Indeed, it is not uncommon for individuals in this phase to neglect personal care and oral hygiene.^20^ Moreover, adolescence is a period in which parents begin to designate more responsibility to their children and diminish their vigilance in certain areas, such as oral health care.^10^

Comparing the results of the second examination to the baseline findings, improvements in oral hygiene were found in both groups, reflecting a change in the behaviour of the adolescents. Thus, information technologies proved to be as effective as oral counseling. This is an important finding, as technological tools enable a constant, direct approach that does not require mediation on the part of parents, dentists or teachers. Moreover, taking an individual-centered approach, in which each person is responsible for maintaining his/her own health, enables better outcomes and a reduction in health expenditures.^34^ Educational actions based on information technologies are effective for the acquisition of better oral health habits, such as the frequent use of dental floss, resulting in lower gingival bleeding indices.

The results of the study should be analysed with caution in terms of its external validity. However, the blinding of the researcher during clinical data collection is an important aspect of this study’s methodological design, as it avoids biased results.

## Conclusion

The present clinical trial demonstrates that the use of information technologies is effective at improving the frequency of dental floss use among adolescents. Thus, the incorporation of such technologies is a valid strategy for health education actions, considering the vast potential of these tools regarding the acquisition of heathy habits, especially by the young population.
